# In Silico SAR Studies of HIV-1 Inhibitors

**DOI:** 10.3390/ph11030069

**Published:** 2018-07-13

**Authors:** Ismail Hdoufane, Imane Bjij, Mahmoud Soliman, Alia Tadjer, Didier Villemin, Jane Bogdanov, Driss Cherqaoui

**Affiliations:** 1 Department of Chemistry, Faculty of Science Semlalia, BP 2390 Marrakech, Morocco; ismail.hdoufane@edu.uca.ma (I.H.); imane.bjij@gmail.com (I.B.); 2 School of Health Sciences, University of KwaZulu-Natal, Westville, Durban 4000, South Africa; Soliman@ukzn.ac.za; 3 Faculty of Chemistry and Pharmacy, 1 James Bourchier Avenue 1164, Sofia University “St. Kliment Ohridski”, Sofia 1164, Bulgaria; tadjer@chem.uni-sofia.bg; 4 Ecole Nationale Supérieure d'Ingénieurs (ENSICAEN) LCMT, UMR CNRS n° 6507, 6 Boulevard Maréchal Juin, 14050 Caen, France; didier.villemin@ensicaen.fr; 5 Institute of Chemistry, Faculty of Natural Sciences and Mathematics, Ss. Cyril and Methodius University, 1000 Skopje, Macedonia; j_b_bogdanov@yahoo.com

**Keywords:** structure activity relationship, TIBO, HIV inhibitors, support vector machines, decision trees, random forests and artificial neural networks

## Abstract

Quantitative Structure Activity Relationships (QSAR or SAR) have helped scientists to establish mathematical relationships between molecular structures and their biological activities. In the present article, SAR studies have been carried out on 89 tetrahydroimidazo[4,5,1-jk][1,4]benzodiazepine (TIBO) derivatives using different classifiers, such as support vector machines, artificial neural networks, random forests, and decision trees. The goal is to propose classification models that will be able to classify TIBO compounds into two groups: high and low inhibitors of HIV-1 reverse transcriptase. Each molecular structure was encoded by 10 descriptors. To check the validity of the established models, all of them were subjected to various validation tests: internal validation, Y-randomization, and external validation. The established classification models have been successful. The correct classification rates reached 100% and 90% in the learning and test sets, respectively. Finally, molecular docking analysis was carried out to understand the interactions between reverse transcriptase enzyme and the TIBO compounds studied. Hydrophobic and hydrogen bond interactions led to the identification of active binding sites. The established models could help scientists to predict the inhibition activity of untested compounds or of novel molecules prior to their synthesis. Therefore, they could reduce the trial and error process in the design of human immunodeficiency virus (HIV) inhibitors.

## 1. Introduction

Human immunodeficiency virus (HIV) is a member of a family of viruses called retroviruses and belongs to a subgroup called lentiviruses. HIV affects and destroys the immune system of the body and causes acquired immunodeficiency syndrome (AIDS) disease. Enzymes responsible for HIV-1 replication have been identified as therapeutic targets. The existing strategies for developing HIV antiviral agents depend mainly on disrupting the replication of these enzymes. Reverse transcriptase (RT) is one of the main targets for antiretroviral drug development due to its essential role in the HIV replication. HIV uses its RT to convert its RNA genome into DNA. Thus, inhibition of this activity impedes HIV’s ability to replicate and to infect additional cells. RT has thus become a subject of considerable pharmaceutical research and a major target of anti-AIDS drug design [1]. A large number of inhibitors have been designed [2], synthesized, and assayed, and some HIV-1 RT inhibitors are now utilized in the treatment of AIDS.

The development of a new drug is a long, tedious, and costly process, ranging from the identification of a biological target of therapeutic interest to the patient, in which clinical trials follow pre-clinical development. Twelve years (often much longer) and nearly one billion dollars are needed from target identification through synthesis, fabrication protocol and testing to approval for marketing [3]. For these reasons, the research laboratories have shown great interest in theoretical methods that enable the rational design of pharmaceutical products. Numerous molecular modelling approaches have been reported addressing the design of new anti-HIV compounds. Most of them are based on QSAR methods. Their objective is to find mathematical relationships between a biological activity of a molecular system and its molecular properties. The QSARs are regarded as efficient tools for the drug development process.

The tetrahydroimidazo[4,5,1-jk][1,4]benzodiazepines (TIBOs), as non-nucleoside analogues, constitute a group of potent inhibitors of HIV-1 RT. This family of compounds was the subject of several 2D and 3D QSAR studies [4,5,6,7,8], and many models have been developed from this database. Research work carried out on this family of compounds treated only correlation problems. To the best of our knowledge, no classification work has been reported on TIBO derivatives using support vector machines (SVM), artificial neural networks (ANN), random forests (RF), and decision trees (DT) except a preliminary and short study that we have published recently, in conference proceedings [9], using SVM and DT on the same compounds that we will treat herein. In continuation of such efforts, our objective is to contribute to the design of HIV inhibitors with reliable classification models. The advantage is the opportunity to build more stable models when biological activity values cannot be determined accurately for a variety of reasons, e.g., lack of sensitivity of a particular test system.

In this work, four chemometric methods (SVM, ANN, DT, and RF) were used. Our goal is to propose classifiers that are able to classify TIBO compounds into two groups: high and low active compounds, and then to find the variables responsible for this classification. The contribution of each descriptor to the model establishment is evaluated.

## 2. Materials and Methods

### 2.1. Selection of Data Set

**Figure 1:**
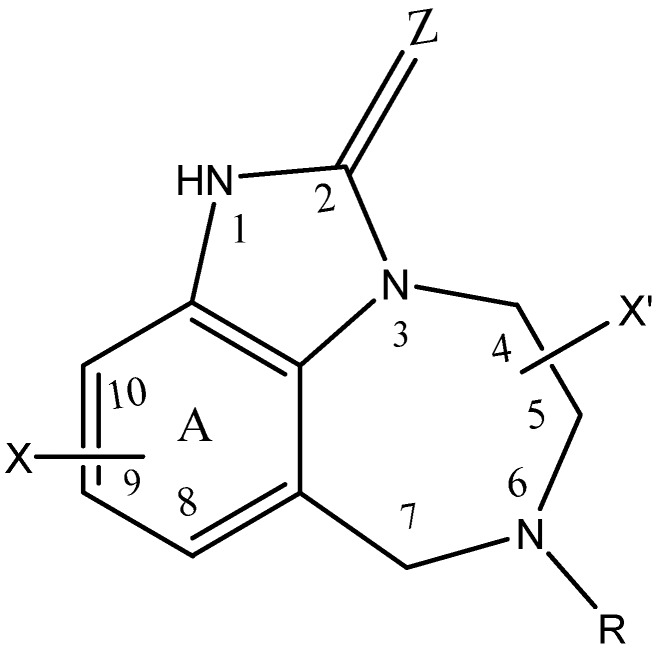
General structure of TIBO derivatives studied.

The data set used in this paper contains 89 TIBO derivatives, which were collected from a published study [10]. These compounds share the same core structure and have different substituents (Figure 1). The anti-HIV activity of the compounds has been expressed by their ability to inhibit the enzyme (RT). The concentration of the compound causing 50% inhibition has been measured and expressed as IC_50_. The endpoint under consideration is the negative decimal logarithm of IC_50_ (pEC_50_ = log (1/IC_50_).

Since it is a classification study (qualitative), the original dependent variable (log (1/IC_50_)) was divided into two classes:

- Class *H* includes compounds with high activities (i.e., log (1/IC_50_) ≥ 5.79).
- Class *L* contains compounds with low activities (i.e., log (1/IC_50_) < 5.79).

The threshold of the division (5.79) represents the average of the minimum and maximum values of the database. Thus, 40 compounds were assigned to class *H* and 49 to class *L*. In Table 1 all compounds with their allocated classes are shown.

The data set (89 compounds) was divided into a training set (69 compounds) and a test set (20 compounds), where the former set is used to develop the classifier and the latter to evaluate its performance. The test set is selected such that each of its members is close to at least one point of the training set (Table 1).

### 2.2. Molecular Descriptors

A molecular descriptor is the result of a mathematical treatment that transforms the chemical information of a molecule into a numerical value. Currently, there are several commercial and free software packages that can calculate a large number of descriptors representing several classes: steric, electronic, topological, hydrophobic, and thermodynamic. To reduce this number, various regression and classification-based methods are used to develop robust QSAR/SAR models [11]. More than 500 molecular descriptors from five classes (geometrical, topological, constitutional, electrostatic, and quantum-chemistry descriptors) were calculated for the present 89 TIBO derivatives [12]. Due to the high number of descriptors considered, the author has used the stepwise MLR (multiple linear regression) procedure to select the powerful variables. To avoid all difficulties in the interpretation of the resulting models, pairs of variables with a correlation coefficient greater than 0.80 were classified as interrelated and only one of these variables was chosen [12]. Seven molecular descriptors were selected [12] and are used in the present study (Table 2). They characterize hydrophobic, electronic, and topological aspects of the molecules. MD in Table 2 stands for molecular descriptor.

Three other descriptors have been added because of their importance for protein-inhibitor interaction [10]:

MD8 = 1 for TIBO compounds having substituent R = 3,3-dimethyallyl and MD8 = 0 when R ≠ 3,3-dimethyallyl (Figure 1).

MD9 = 1 if Z = Sulphur and MD9 = 0 if Z = Oxygen (Figure 1).

The X-substituents can occupy three different positions at ring A (Figure 1). All of them are shown to contribute to the RT inhibition by their hydrophobic effect. However, The X-substituents at the 8-position have a steric effect as well [10]. For all these reasons, a tenth descriptor (MD10) has been added such as its value is equal to 1, 0.5, or 0 for compounds with an X-substituent in the 8, 9, or 10 position, respectively.

## 3. Results and Discussion

In this work, SVM, ANN, DT, and RF were used as machine learning algorithms and five different sessions have been achieved: computation and internal validation, Y-randomization, prediction, the descriptor’s contribution, and molecular docking analysis. The first one was used not only for the parameterization of each chemometric technique, but also for evaluating the internal validation of the established models. The leave one out (LOO) cross validation procedure [13] has been used for this internal validation. The second session was performed to ensure the robustness of the developed SAR models. The third one was aimed at determining the predictive ability of the established models. In the fourth session, we attempt an evaluation of the importance of the descriptors used. In addition, the applicability domain has been defined to assess the prediction by interpolation of established models. Molecular docking analysis was carried out in the last session, to study the interactions between the reverse transcriptase enzyme and the TIBO compounds studied.

### 3.1. Support Vector Machines

SVM are supervised learning algorithms that can be applied to classification or regression problems. SVM algorithms were mainly developed by Vapnik [14] and have been used in a range of problems, including pattern recognition, bioinformatics, and text categorization. Several books and research papers deal with the theory of SVM [11,15], and a brief outline of its description and its application for classification purposes is given below.

SVM are learning algorithms initially built for binary classification. The idea is to look for a decision rule based on an optimal margin hyperplane separation. There are two cases: the training dataset is linearly separable or non-linearly separable. The first case is very simple and can be solved by several classifiers, including SVM. However, many problems are not linearly separable. Therefore, the extension of the SVM to the second case relies on the projection of the input data into a higher dimensional space where a linear hyperplane can easily be found. In this case, SVMs introduce the notion of a kernel induced feature space, which projects the data into a higher dimensional space where the data is separable. The choice of a kernel function influences the classification performance of a SVM. Many kernel function formulations have been proposed in the literature [16], including linear, polynomial, and radial basis function (RBF). However, for classification tasks, a commonly used kernel function is the RBF because of both its good generalization performance and its just two parameters that have to be optimized [17]. Therefore, we used this kernel function for the SVM classification model and its formula is defined as follows:

(1) $$\exp(-\gamma {\left\|\mu -\nu \right\|}^{2})$$

where *γ* is the parameter of the kernel, and *μ* and *ν* are two independent variables.

The choice of appropriate learning parameters is a crucial step in obtaining good classification models. In SVM classification problems, the parameters to be optimized are: *C*, the parameter that controls the trade-off between maximizing the margin and minimizing the training error, and *γ* the parameter of the RBF. The settings of the *C* and *γ* parameters are based on a so-called grid search. For the present study, the selection of these parameters was done by a grid-search of LIBSVM [18]. The range of the grid-search for log_2_(*C*) was [−5, 15] with a step size of two and the one for log_2_(*γ*) was [−5, 1] also with an increment of two. For each of the parameter pairs (*C*, *γ*), a five-fold cross validation was performed. In the five-fold cross validation, one-fifth of the data is held out for testing and the remaining four-fifths are used for training. This is iterated five times and the evaluation metrics are averaged across the five iterations. After the grid search was done, the parameter pair, (*C*, *γ*), at which the cross-validation had the highest correct classification rate (CCR) was (8, 0.5). These parameters were later used to build the final classifier model.

Using *C* = 8 and *γ* = 0.5, the CCR of the training set was 100% (All 69 compounds were well classified). The CCR on LOO cross validation was 92.75%. The randomization test (Y-randomization) is a useful additional tool used in the validation of QSAR/SAR models. This procedure ensures that the model is not due to chance [19]. The *Y* column [*Y* = log (1/IC_50_)] is randomly shuffled and a new classification SAR model is developed using the original independent molecular descriptors matrix. The newly established randomized SAR models are expected to have lower CCR values compared to those of the original non-randomized one. In this study, five random shuffles of the *Y* column were performed using LOO. The average CCR was 60.90%. This value was much lower than the one obtained for the real model (92.75%) and, thus, it excluded the possibility of a chance correlation.

A test set of compounds, which have not been taken into consideration in the training set, was used to estimate the predictive power of SVM. The model constructed from the 69 compounds was used to predict the classes of the ones contained in the test set (Table 1). The CCR obtained was 85% (Compounds **26**, **30,** and **32** were misclassified (see Table 1)).

### 3.2. Artificial Neural Networks

ANN (also known as neural networks) are computational models simulating the function of the human brain. The use of ANN has been developed in many disciplines (economics, ecology and environment, chemistry, biology, and medicine, etc.). They are applied to solve problems of classification, prediction, optimization, and pattern recognition. Three components constitute a neural network: The processing elements or nodes, the topology of the connections between the nodes, and the learning rule by which new information is encoded in the network. Therefore, various ANN models are given in the literature, like Hopfield’s network, Kohonen’s network, and the multilayer perceptron [20]. However, the most commonly used and successful ANN in QSAR/SAR studies is the three-layered feed-forward network [21]. In this type of network, three layers are interconnected. The first layer consists of input neurons. Those neurons send data to the second layer (hidden layer), which in turn sends the output neurons to the third layer (output layer). Through an iterative process (called the back-propagation algorithm), the connection weights are modified until the network gives the desired results for the training set of data. We described this algorithm with a simple example of an application [22], and a detailed description of this algorithm is given elsewhere [23].

In this work, a three-layered ANN was used too. The neurons were arranged in layers (the input, hidden, and output layers), in which:

✓ The input layer contains ten neurons, representing the ten parameters described previously;
✓ The output layer contains a single neuron describing the class of the compound (Low or High HIV inhibitor)
✓ The hidden layer contains a variable number of neurons. This layer allows ANN to model nonlinear relationships between inputs and outputs.

In the application of ANN, the step that seems to be the most difficult is the determination of the architecture of the network. Indeed, the numbers of the neurons of the input and output layers are known. However, there is no precise and rigorous method for determining the optimal number of hidden neurons. Having too many hidden neurons may lead to an overtraining of the ANN. On the other hand, fewer hidden neurons will not give sufficient information to the ANN to accurately learn the relationship between the input and output layers. Some studies [24,25] have proposed a parameter defined as the ratio between the number of compounds in the training set and the number of connections in the ANN. To extract all the relevant features and to give good predictions, it was suggested that this parameter should be between 1 and 3 [24,25].

Using the WEKA software [26], we have varied the number of hidden neurons to maintain the value of this parameter between 1 and 3. Four ANN architectures of 10-*x*-1 (*x* = 3–6, *x* represents the number of hidden neurons) have been tested for the training procedure. The number of cycles was limited to 1000. Among all architectures of the ANN, the best one was 10-5-1. The CCR was 98.55% (only compound **89** was misclassified). We have tried two hidden layers with a different number of hidden neurons, but the best result is the one obtained with a single hidden layer containing five neurons (10-5-1 ANN architecture). Applying this architecture, the CCRs were 92.75% and 53.60% for the internal validation and Y-randomization, respectively. These results are encouraging and show that the 10-5-1 established model is not obtained by chance. After attaining these interesting results, we have tested its predictive power on the test set and the CCR was 90% (compounds **30** and **83** were misclassified (see Table 1)).

### 3.3. Decision Trees (DT) and Random Forest (RF)

The decision tree is one of the most supervised learning classification algorithms used in data mining and pattern recognition. A DT is a classifier in the form of a tree structure that begins with a single root node at which a condition is verified about the element to be classified. Each possible answer leads to another node, which may be either an internal node, at which another condition is verified, or a leaf node, which has no children and at which the classification is given. Many classification and DT generation methods have been researched. C4.5 is one of the most effective algorithms developed by Quinlan [27] and is used to generate a DT. DTs produced by the C4.5 algorithm depend essentially on two parameters: the pruning confidence factor (C) and minimum number of split-off cases (M).

**Figure 2:**
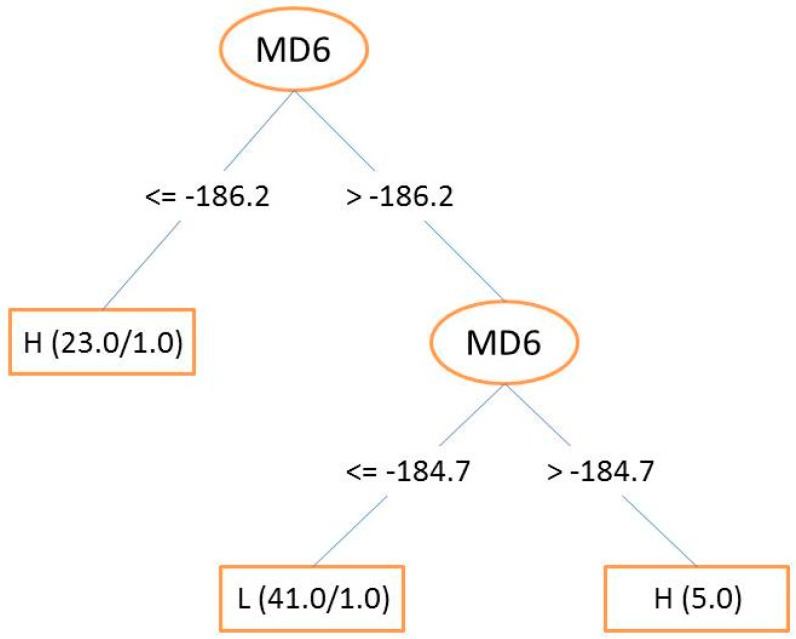
Decision tree for TIBO derivatives.

In this section, the used approach consists in building a decision tree by using the J48 classification tool, implemented in the WEKA software [28] (C4.5 is termed as J48 in this software). The objective is to build a decision tree able to classify the TIBO compounds into class “*H*” or “*L*”. The default values of *C* and *M*, for a J48 decision tree in WEKA, are 0.25 and 2, respectively. In this study, several combinations of *C* and *M* values have been tested, but the classification results found are the same. Therefore, these parameters were kept constant to the default values (C = 0.25 and M = 2). From the 69 molecules of the training set, the tree shown in Figure 2 has been built. From this figure, we can observe that only one descriptor, MD6, was enough to build this classification model. Only two molecules, 60 and 65, were misclassified. The CCR value for the training set was 97.10% (96.43% for *H* compounds and 97.56% for *L* compounds). To ensure the robustness of this developed model, internal validation and Y-randomization were performed using the default values of *C* and *M*. The CCRs obtained were 92.75% and 49.30% for internal validation and Y-randomization, respectively. These results show that the model is reliable. The predictive power of this DT was then applied to the test set and the resulting CCR was 70% (66.67% and 75% for *H* and *L* compounds, respectively). Four *H* compounds (**30**, **66**, **67**, and **81**) and two *L* compounds (**26** and **32**) were misclassified (see Table 1).

Instead of using a single decision tree, we thought it would be useful to apply multiple decision trees at the same time. That is why we have used the RF algorithm [29] to study this classification problem. The RFs are a set of decision trees for building predictive models. They are popular for machine learning tasks and are mostly used in classification problems. Each tree produces a response when presented with a set of predictor values. An RF consists of an arbitrary number of simple trees that will lead to a final response. For classification problems, the ensemble of these trees allows the choosing of the most popular class. In other words, the classification having the most votes is chosen by the forest. In the present study, the CCRs obtained by the RF were 100%, 95.65%, and 75% for the training set, internal validation, and test set, respectively. All the compounds in the training set were well-classified. However, for the test set, three *H* compounds (**30**, **81**, and **83**) and two *L* compounds (**26** and **32**) were misclassified (see Table 1).

### 3.4. Comparison between ANN, DT, SVM, and RF

**Figure 3:**
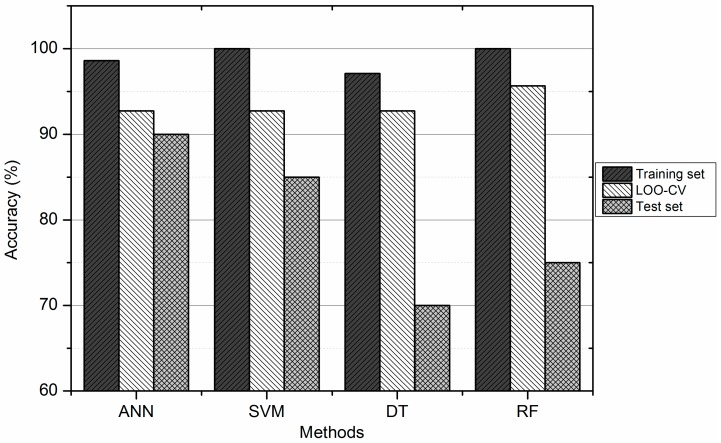
Correct Classification Rate (CCR) of SVM, ANN, DT, and RF.

The results obtained with the four different methods are summarized in Table 3 and are shown in Figure 3. As we can see from Table 3, columns 3 and 6 give the sensitivity of each model for the training and test sets, respectively. According to these results, all these classifiers were able to establish a satisfactory relationship between the molecular descriptors and the anti-HIV activity. However, one of the most important features of a SAR model is its ability to accurately predict the classes of compounds that were not used for the model development (test set). That is the reason we consider that ANN and SVM had better predictive power than DT and RF. The flexibility of SVM and ANN enables them to establish complex nonlinear relationships in the experimental data. Furthermore, ANN is able to extract the necessary information from examples, without explicitly incorporating rules into the network, to find relationships between molecular structures and their biological activities. DTs and RFs tend to overfit on training data. This leads sometimes to poor performance on unseen data.

In Table 4, the full list of misclassified compounds by SVM, ANN, DT, and RF for the training and test sets are shown. It is difficult, if not impossible, to find the reason why the model failed to predict them accurately. Misclassified compounds, by one or two methods, could come from the prediction limits of each classifier. However, we were interested in understanding why among them some compounds were misclassified by three or four methods. Compound 30 was misclassified by all methods and compounds **26** and **32** were misclassified by SVM, DT, and RF. The applicability domain is one of the methods used to understand these misclassified compounds [30]. It provides useful information to explain why some compounds are classified with good accuracy and others are expected to have poor and unreliable predictions. In this study, the applicability domain was applied to the test set using the leverage method [31]. Two compounds, **24** and **30**, have been outside this domain. This method showed that compound 24 was close to the applicability domain, while compound **30** was far from it.

The values of the inhibitory activity of the compounds, **26** and **32** (5.61 and 5.33 for compounds **26** and **32**, respectively), which are close to that taken as a reference (5.79) can explain the inability of the model to accurately predict the classes of these two compounds.

### 3.5. Descriptor Contributions

The contribution of a molecular descriptor to the QSAR/SAR models provides good insights into the forces controlling the activity of the compounds. Various methods have been proposed for evaluating the importance of each independent input parameter in determining a dependent output response. In the current work, the contribution of each descriptor was calculated using the following methods [32]: Info Gain Attribute Eval-Ranker, Gain Ratio Attribute Eval-Ranker, and Symmetrical Uncert Attribute Eval-Ranker. The average values of these four methods were calculated (Table 5).

Table 5 indicates that the relative importance of the descriptors (average values) varied in the following order: MD6 > MD3 > MD1 > MD8 > MD5 > MD7 > MD9 > MD10 > MD4 > MD2.

This result shows that all the descriptors, except MD2, contribute to the inhibition of HIV replication. We have re-performed all previous calculations without taking into account the MD2 descriptor and we have found the same classification results. This means that the MD2 descriptor does not contribute to the SAR model establishment. However, the contributions of the other nine descriptors show their importance in the inhibition of the reverse transcriptase by the TIBO compounds. Thus, these descriptors, encoding significant structural information, such as electronic, steric, and hydrophobic effects, can be used to establish reliable anti-HIV-1 SAR models. This confirms the results of our [6] and other authors’ [10] studies according to which the HIV-1 reverse transcriptase inhibition activity of the TIBO compounds is mainly governed by these three effects.

### 3.6. Molecular Docking Study

For a better comprehension of the SAR classification findings, a molecular docking study was carried out using the Lamarkian Genetic algorithm within the Autodock Vina software [33]. The TIBO derivatives used in this study, along with the reverse transcriptase, were prepared using AutoDock Tools GUI [34]. A semi-flexible docking protocol was applied, where the receptor was considered as a rigid unit. However, translation and rotation of the ligands in the gridbox and rotation around single bonds were allowed. A docking gridbox was generated to describe the catalytic site of the enzyme using a grid point spacing of 0.266 Å with dimensions of 30 × 30 × 30 (Å). This gridbox was defined with *X*, *Y*, and *Z* centers as 3.019, −38.021, and 23.005, respectively. The results were analyzed using the Discovery studio 2018 [35] and Maestro from the Schrodinger suite [36].

The crystal structure of HIV-1 reverse transcriptase (HIV-1/RT) is complexed with different ligands and is available in the Protein Data Bank (PDB). This protein involves a valuable active site that can be useful in the design of potent inhibitors. Since our study addresses TIBO derivatives, we have exploited the structure of HIV-1/RT complexed with 9-chloro-TIBO (TB9) (PDB entry code: 1REV) [37]. The crystalized ligand (TB9) was removed and then all compounds from our dataset were docked in the binding site of the studied receptor.

**Figure 4:**
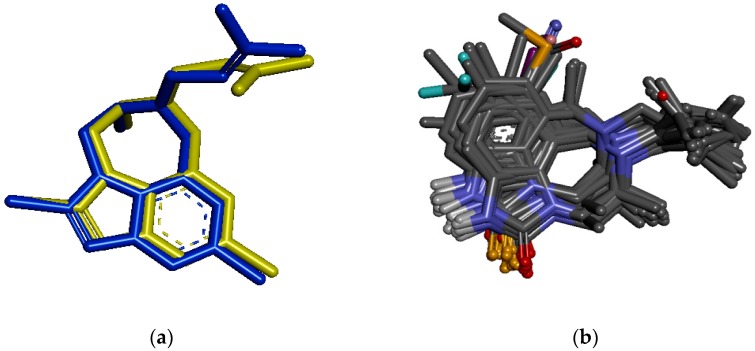
(**a**): Alignment view of the pre-existing (blue) and docked (yellow) ligands. (**b**): Alignment view of high and low TIBO derivatives.

Prior to the docking of our dataset into the binding site of the macromolecule, we attempted to re-dock the crystalized ligand, TB9, in the same binding site of 1REV to verify the docking process. Figure 4a shows the alignment view of the docked and the pre-existing TB9 ligands. A root mean square deviation (RMSD) of 0.279 Å between these two ligands validates the docking protocol.

To gain a better insight into the binding mechanism, all TIBO derivatives were docked in the 1REV receptor. The result indicates that the ligands adopt similar binding orientation as depicted in Figure 4b. We expected to observe this similar orientation because the TIBO compounds have the same main structure. However, on the basis of the docking analyses, we have found differences in the binding affinity between high and low active compounds.

**Figure 5:**
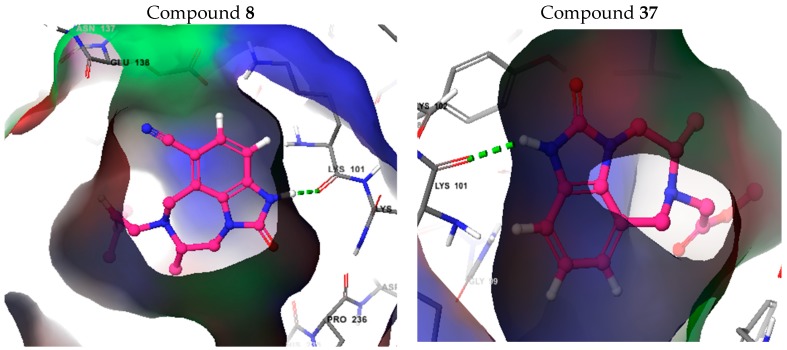
Hydrogen bond between the imidazolone ring of the ligand and the LYS101 residue of the RT. Hydrophobic, electrostatic, and steric contour maps are represented by green, blue, and red contours, respectively.

**Figure 6:**
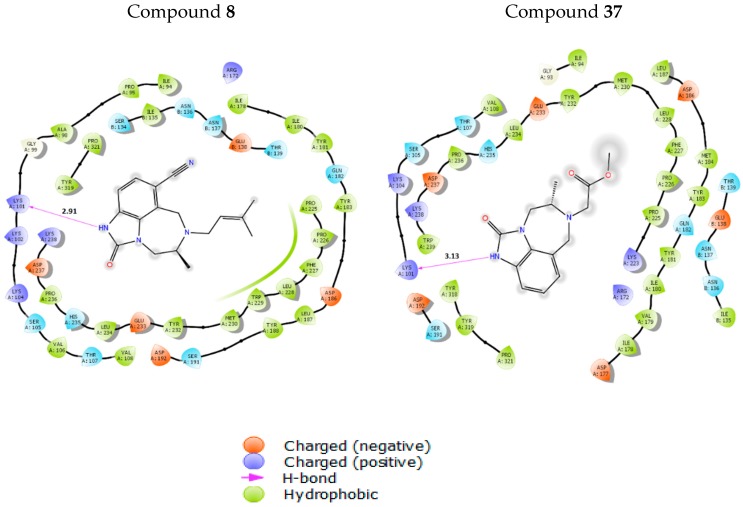
Hydrogen bond, hydrophobic, and electrostatic interactions as exhibited by compounds 8 (**left**) and 37 (**right**).

Compounds **8** (having the highest affinity) and **37** (having the lowest affinity) were chosen to illustrate the ligand-receptor interactions and to understand the phenomena involved in the inhibition of the RT. Hydrogen bonding interactions, similar to those of TB9, have been observed for both compounds **8** and **37**. Indeed, one hydrogen bond is formed between the imidazolone ring of the ligand and the LYS101 residue of the receptor binding site (Figure 5). The hydrogen bonding distance of compound 8 (2.91 Å) is shorter than that of compound **37** (3.13 Å) (Figure 6) and, thus, this effect contributes better to the affinity between compound **8** and the receptor (RT). This interaction could also explain the importance of the MD3 and MD5 parameters in the previously established models. Moreover, the affinity of TIBO compounds is shown to be governed by the hydrophobic interactions, in particular, compounds with a DMA group at the 6N position of the diazepine ring, and this might be the reason for the high activity of ligands containing a DMA group (like compound 8). Indeed, on the one hand, the 1REV/RT active site involves MET230, TRP229, LEU228, PHE227, PRO226, PRO225, TYR188, LEU187, and TYR183 amino acid residues in the binding interactions with TIBO compounds having a DMA group at site-6 of the diazepine ring along with TYR319, PRO321, ALA98, and PRO95 with the phenyl ring (Figure 6). On the other hand, the calculated MD1 descriptor (log P) shows that compounds with DMA as an R-group have the highest value of log P (including compound 8 with a value of 2.00), while compound 37 with a [CH_2_CO_2_Me]-substituent has the lowest value of log P (0.26). Small values of log P are an indication of hydrophilicity and a loss of hydrophobicity. This confirms not only the importance of the MD1 descriptor, but also of the MD8 descriptor, which indicates the presence or absence of a DMA group at the 6N position of the diazepine ring. The nitrogen atom at this position and the other one in the imidazolone ring, engaged in the hydrogen bond, play an important role in the affinity between the ligand and the RT, and, thus, could explain the importance of the MD4 and MD6 descriptors. Furthermore, the binding pocket surface maps (Figure 5 and Figure 6) show relative electronic and steric interactions.

## 4. Conclusions

In the present work, four methods, SVM, ANN, DT, and RF, were used to develop SAR models of anti-HIV-1 TIBO derivatives. All established models show correct and reliable classification rates. The comparison between these methods, assessed using an external validation test set, demonstrates that the performances of ANN and SVM models are better than those of DT and RF. ANN and SVM are very efficient tools for classification problems and they can be successfully used in QSAR/SAR applications.

The molecular descriptor contribution analysis showed that the electronic, steric, and hydrophobic parameters are the main factors controlling the activity of TIBO derivatives. This information can be taken into account in the design of new HIV-1 inhibitors. The established classification models can be used in biological screening processes and in the prediction of the inhibition of HIV-1 reverse transcriptase of untested molecules.

## Figures and Tables

**Table 1:** Chemical structures of the compounds studied and their anti-HIV activity

Substituents						Classes			
N	X	Z	R	X’	^a^ Exp	^b^ SVM	^c^ ANN	^d^ DT	^e^ RF
1	H	S	DMA	5-Me(*S*)	H	H	H	H	H
2	9-Cl	S	DMA	5-Me(*S*)	H	H	H	H	H
^t^ 3	8-Cl	S	DMA	5-Me(*S*)	H	H	H	H	H
4	8-F	S	DMA	5-Me(*S*)	H	H	H	H	H
5	8-SMe	S	DMA	5-Me(*S*)	H	H	H	H	H
^t^ 6	8-OMe	S	DMA	5-Me(*S*)	H	H	H	H	H
7	8-OC_2_H_5_	S	DMA	5-Me(*S*)	H	H	H	H	H
8	8-CN	O	DMA	5-Me(*S*)	H	H	H	H	H
^t^ 9	8-CN	S	DMA	5-Me(*S*)	H	H	H	H	H
10	8-CHO	S	DMA	5-Me(*S*)	H	H	H	H	H
11	8-CONH_2_	O	DMA	5-Me(*S*)	L	L	L	L	L
12	8-Br	O	DMA	5-Me(*S*)	H	H	H	H	H
^t^ 13	8-Br	S	DMA	5-Me(*S*)	H	H	H	H	H
14	8-I	O	DMA	5-Me(*S*)	H	H	H	H	H
^t^ 15	8-I	S	DMA	5-Me(*S*)	H	H	H	H	H
16	8-C=CH	O	DMA	5-Me(*S*)	H	H	H	H	H
^t^ 17	8-C=CH	S	DMA	5-Me(*S*)	H	H	H	H	H
18	8-Me	O	DMA	5-Me(*S*)	H	H	H	H	H
19	8-Me	S	DMA	5-Me(*S*)	H	H	H	H	H
20	9-NO_2_	O	CPM	5-Me(*S*)	L	L	L	L	L
^t^ 21	8-NH_2_	O	CPM	5-Me(*S*)	L	L	L	L	L
22	8-NMe_2_	O	CPM	5-Me(*S*)	L	L	L	L	L
23	9-NH_2_	O	CPM	5-Me(*S*)	L	L	L	L	L
^t^ 24	9-NMe_2_	O	CPM	5-Me(*S*)	L	L	L	L	L
25	9-NHCOMe	O	CPM	5-Me(*S*)	L	L	L	L	L
^t^ 26	9-NO_2_	S	CPM	5-Me(*S*)	L	H	L	H	H
27	9-F	S	DMA	5-Me(*S*)	H	H	H	H	H
28	9-CF_3_	O	DMA	5-Me(*S*)	L	L	L	L	L
^t^ 29	9-CF_3_	S	DMA	5-Me(*S*)	H	H	H	H	H
^t^ 30	9-Me	O	DEA	5-Me(*S*)	H	L	L	L	L
31	10-OMe	O	DMA	5-Me(*S*)	L	L	L	L	L
^t^ 32	10-OMe	S	DMA	5-Me(*S*)	L	H	L	H	H
33	9,10-di-Cl	S	DMA	5-Me(*S*)	H	H	H	H	H
34	10-Br	S	DMA	5-Me(*S*)	H	H	H	H	H
35	H	O	CH_2_CH=CH_2_	5-Me(*S*)	L	L	L	L	L
36	H	O	2-MA	5-Me(*S*)	L	L	L	L	L
37	H	O	CH_2_CO_2_Me	5-Me(*S*)	L	L	L	L	L
^t^ 38	H	O	CH_2_C≡CH	5-Me(*S*)	L	L	L	L	L
39	H	O	CH_2_-2-furanyl	5-Me(*S*)	L	L	L	L	L
40	H	O	CH_2_CH=CH_2_[S(+)]	5-Me(*S*)	L	L	L	L	L
41	H	O	CH_2_CH_2_CH=CH_2_	5-Me(*S*)	L	L	L	L	L
42	H	O	CH_2_CH_2_CH_3_	5-Me(*S*)	L	L	L	L	L
43	H	O	2-MA[S(+)]	5-Me(*S*)	L	L	L	L	L
44	H	O	CPM	5-Me(*S*)	L	L	L	L	L
^t^ 45	H	O	CH_2_CH=CHMe(*E*)	5-Me(*S*)	L	L	L	L	L
46	H	O	CH_2_CH=CHMe(*Z*)	5-Me(*S*)	L	L	L	L	L
47	H	O	CH_2_CH_2_CH_2_Me	5-Me(*S*)	L	L	L	L	L
48	H	O	DMA	5-Me(*S*)	L	L	L	L	L
49	H	O	CH_2_C(Br)=CH_2_	5-Me(*S*)	L	L	L	L	L
50	H	O	CH_2_C(Me)=CHMe(*E*)	5-Me(*S*)	L	L	L	L	L
51	H	O	DMA[*R*(+)]	5-Me(*S*)	L	L	L	L	L
52	H	O	DMA[*S*(+)]	5-Me(*S*)	L	L	L	L	L
^t^ 53	H	O	CH_2_C(C_2_H_5_)=CH_2_	5-Me(*S*)	L	L	L	L	L
54	H	O	CH_2_CH=CHC_6_H_5_(*Z*)	5-Me(*S*)	L	L	L	L	L
55	H	O	CH_2_C(CH=CH_2_)=CH_2_	5-Me(*S*)	L	L	L	L	L
56	8-Cl	S	DMA	H	H	H	H	H	H
57	9-Cl	S	DMA	H	H	H	H	H	H
58	H	O	2-MA	5,5-di-Me	L	L	L	L	L
59	H	O	2-MA	4-Me	L	L	L	L	L
60	9-Cl	S	2-MA	4-Me(*S*)	H	H	H	L	H
61	9-Cl	S	CPM	4-Me(*R*)	L	L	L	L	L
62	H	O	C3H7	4-CHMe_2_	L	L	L	L	L
63	H	O	2-MA	4-CHMe_2_	L	L	L	L	L
64	H	O	2-MA	4-C_3_H_7_	L	L	L	L	L
65	H	O	DMA	7-Me	L	L	L	H	L
^t^ 66	8-Cl	O	DMA	7-Me	H	H	H	L	H
^t^ 67	9-Cl	O	DMA	7-Me	H	H	H	L	H
68	H	S	C_3_H_7_	7-Me	L	L	L	L	L
69	H	S	DMA	7-Me	H	H	H	H	H
70	8-Cl	S	DMA	7-Me	H	H	H	H	H
71	9-Cl	S	DMA	7-Me	H	H	H	H	H
72	H	O	DMA	4,5-di-Me(*cis*)	L	L	L	L	L
73	H	S	DMA	4,5-di-Me(*cis*)	L	L	L	L	L
^t^ 74	H	S	CPM	4,5-di-Me(*trans*)	L	L	L	L	L
75	H	S	DMA	4,5-di-Me(*trans*)	L	L	L	L	L
76	H	S	DMA	5,7-di-Me(*trans*)	H	H	H	H	H
77	H	S	DMA	5,7-di-Me(*cis*)	H	H	H	H	H
78	9-Cl	O	DMA	5,7-di-Me(*R,R-trans*)	H	H	H	H	H
79	9-Cl	S	DMA	5,7-di-Me(*R,R-trans*)	H	H	H	H	H
80	H	S	DMA	4,7-di-Me(*trans*)	L	L	L	L	L
^t^ 81	9-Cl	O	DMA	5-Me(*S*)	H	H	H	L	L
82	9-Cl	S	CPM	5-Me(*S*)	H	H	H	H	H
^t^ 83	H	S	CPM	5-Me(*S*)	H	H	L	H	L
84	H	O	C_3_H_7_	5-Me	L	L	L	L	L
85	H	S	C3H7	5-Me	L	L	L	L	L
86	H	O	2-MA	5-Me	L	L	L	L	L
87	H	S	DMA	5-Me	H	H	H	H	H
88	H	O	DMA	5-Me(*S*)	L	L	L	L	L
89	H	S	2-MA	5-Me(*S*)	H	H	L	H	H

^a^ Experimental activity. ^b–e^ Predicted classes by SVM, ANN, DT, and RF, respectively. ^t^ Test set. DMA: 3,3-Dimethylallyl. MA: Methylallyl. CPM: Cyclopropylmethyl. DEA: Diethylallyl.

**Table 2:** List of the selected molecular descriptors and their physical and chemical meanings.

Descriptors	Chemical Meaning
MD1	logP: Octanol/water partition coefficient for the compound studied
MD2	Average nucleophilic reaction index for a N atom
MD3	Minimum total interaction for a H-N bond
MD4	Minimum (>0.1) bond order of a N atom
MD5	ESP-HBSA H-bonding surface area
MD6	Maximum atomic state energy for a N atom
MD7	^3^χ: Molecular connectivity index to the third order

**Table 3:** Classification results of the training and the test sets obtained by the four methods. Sn(H) and Sn(L) are Sensitivity and Specificity, respectively.

Methods	Training Set (%)			Test Set (%)		
	Total Accuracy	*Sn(H)*	*Sn(L)*	Total Accuracy	*Sn(H)*	*Sn(L)*
SVM	100.00	100.00	100.00	85.00	91.67	75.00
ANN	98.55	96.43	100.00	90.00	83.33	100.00
DT	97.10	96.43	97.56	70.00	66.67	75.00
RF	100.00	100.00	100.00	75.00	75.00	75.00

**Table 4:** Misclassified samples by SVM, ANN, DT, and RF.

Method	Sets	Misclassified Compounds
SVM	Training set	
	Test set	26,30,32
ANN	Training set	89
	Test set	30,83
DT	Training set	60,65
	Test set	26,30,32,66,67,81
RF	Training set	
	Test set	26,30,32,81,83

**Table 5:** Contribution of molecular descriptors to the anti-HIV SAR study. Average values are mentioned in the bottom of the table.

MD1	MD2	MD3	MD4	MD5	MD6	MD7	MD8	MD9	MD10
*Info Gain Attribute Eval-Ranker (%)*									
12.96	0.00	13.88	6.70	10.20	22.18	8.80	8.66	8.80	7.82
*Gain Ratio Attribute Eval-Ranker (%)*									
11.44	0.00	10.55	6.21	11.06	18.91	9.78	14.09	9.60	8.35
*Symmetrical Uncert Attribute Eval-Ranker (%)*									
12.02	0.00	13.63	6.34	10.70	20.52	9.35	10.01	9.27	8.15
*Average (%)*									
**12.14**	**0.00**	**12.69**	**6.42**	**10.65**	**20.54**	**9.31**	**10.92**	**9.22**	**8.11**
